# Diabetic ketoacidosis presentations in a low socio-economic area: are services suitable?

**DOI:** 10.1186/s12913-021-06715-7

**Published:** 2021-07-10

**Authors:** Steven James, Kylie Annetts, Thuy Frakking, Marc Broadbent, John Waugh, Lin Perry, Julia Lowe, Sean Clark

**Affiliations:** 1 University of the Sunshine Coast, Petrie, Queensland Australia; 2 University of Melbourne, Parkville, Victoria Australia; 3 Caboolture Hospital, Caboolture, Queensland Australia; 4 University of Queensland, St. Lucia, Queensland Australia; 5 University of Technology Sydney, Ultimo, New South Wales Australia; 6 South Eastern Sydney Local Health District, Prince of Wales Hospital, Randwick, New South Wales Australia; 7 University of Newcastle, Callaghan, New South Wales Australia

**Keywords:** Continuous glucose monitoring, Diabetes mellitus, type 1, Diabetes services, Diabetic ketoacidosis, Healthcare professionals, Telephone, Type 1 diabetes, Socio-economic, Support

## Abstract

**Background:**

Diabetic ketoacidosis causes a significant number of hospitalisations worldwide, with rates tending to increase with remoteness and socioeconomic disadvantage. Our study aimed to explore healthcare professionals’ perceptions of factors affecting presentation of people with type 1 diabetes in a low socioeconomic area of Queensland, Australia.

**Methods:**

This was a qualitative study. Individual semi-structured face-to-face or telephone interviews were completed with patients with type 1 diabetes who had presented in diabetic ketoacidosis, and healthcare professionals who have experience in related care. Data were analysed using Gibbs’s framework of thematic analysis.

**Results:**

Four patients with type 1 diabetes and 18 healthcare professionals were interviewed. Restricted access was identified as a factor contributing to diabetic ketoacidosis and delayed presentation, with ketone testing supplies, continuous glucose monitoring technology and transport considered barriers. Many of these factors were arguably preventable. Opportunities to improve the care available to patients with type 1 diabetes were detailed, with particularly strong support for dedicated out of hours telephone help lines for adults with type 1 diabetes.

**Conclusions:**

Gaps in support for patient self-care to avoid diabetic ketoacidosis presentations and prevent late presentation of diabetic ketoacidosis revealed by this study require service reconfiguration to support care delivery. Until change is made, people with type 1 diabetes will continue to make both avoidable and delayed, acutely unwell, presentations to Emergency Departments.

**Supplementary Information:**

The online version contains supplementary material available at 10.1186/s12913-021-06715-7.

## Background

Type 1 diabetes (T1D) incidence is increasing world-wide, but the cause remains unclear [1]. Australia has one of the highest rates of T1D in the world; half of those diagnosed are aged 18 years or younger [2], with some variations across ethnic groups and geographical areas. High rates of T1D in children and young people means potentially greater numbers developing and progressing disease complications at earlier ages, and ultimately increased healthcare service usage and premature mortality [3].

Good glycaemic control has been well documented to reduce micro and macro-vascular complication onset and progression in people with T1D. Achievement of glycaemic control and maintenance of normal carbohydrate, protein and fat metabolism requires ongoing administration of insulin. The introduction of newer insulin analogues during the past 15 years has afforded increased opportunities for targeted blood glucose management.

However, insufficient insulin administration may result in the development of hyperglycaemia leading to diabetic ketoacidosis (DKA) which, if not treated urgently, can progress to coma, acute kidney failure, cerebral injury or even death [4]. Recognising the importance of preventing DKA, all patients with T1D and their carers should, where applicable and in consultation with their preventative diabetes healthcare team, be provided with a personalised DKA (sick-day) management plan [5, 6]. Despite this, there may be times where such plans are unavailable, poorly understood by patients, or ineffective. Considering the potentially devasting consequences of DKA, it is therefore vital that all people with T1D have access to timely acute care advice, regardless of time or day.

Reasons for insufficient insulin administration are multi-factorial, and include incorrect insulin dosing, ill-health and stress, body image issues and difficulties around access to insulin. Whilst access issues are commonly thought of as only problems in third-world countries, insulin affordability is also of concern to people with T1D in the developed world, regardless of age [7].

Across Australia, DKA is the cause of a significant number of hospitalisations. Nationally, in 2014–15, there were 7132 hospitalisations with a primary diagnosis of DKA [8]. Most (84%) hospitalisations were for people with T1D, and of these, half (54%) were for children and young people aged less than 25 years. Between 2009 and 10 to 2014–15, the number of hospitalisations across Australia for DKA among children and young people increased by 14% (*n* = 2841 vs *n* = 3245), placing a heightened burden on tertiary healthcare services and associated healthcare professional staff, and arguably illustrating health and social system failure. Mean length of hospital stay among children and young people with T1D for DKA is 2.9 days, with mean length of stay for children being longer than for young people (3.5 vs 2.6 days, respectively) [8]. Rates of DKA hospitalisation also tend to increase both with remoteness and socioeconomic disadvantage, with rates in the lowest socioeconomic group 2.4 times that of the highest group [8]. This has been linked to lower income and lack of insurance, amongst other factors [9].

Our study aimed to explore the perceptions of patients with T1D and healthcare professionals of factors affecting presentation of people with T1D in DKA in a local socio-economic area of metropolitan Queensland, Australia; to examine what possible interventions may improve the quality of care for people with T1D who develop DKA; and how best to prevent DKA in this population.

## Methods

This was a qualitative study conducted in Caboolture, widely regarded as being an area of socio-economic disadvantage; confirmed in 2016 through the Socio-Economic Indexes for Areas score [10, 11]. Diabetes support for adults with T1D was provided through a local diabetes education service operating during standard business hours; no access to after-hours specialist diabetes support was provided. Data were collected by individual semi-structured face-to-face or telephone interviews during March 2020 to March 2021. Maximum variation sampling was used to obtain a broad cross section of potential respondents: patients with T1D and key healthcare professionals with experience in the care of people with T1D presenting in DKA at a designated hospital. Patients and healthcare professional members were recruited following response to posters for the study, advertised across the hospital.

The interview schedule contained open questions (Additional file 1), developed through discussion by research team members following review of available literature, review by expert clinicians involved in the care of people with T1D and piloted with healthcare professionals external to the hospital. This process had resulted in modification of some questions.

Following provision of written informed consent and in line with COVID-Safe recommendations, interviews were held either face to face in a private office or via telephone. Patient recruitment was undertaken until a defined date, whereas healthcare professionals were recruited until data saturation was achieved. All interviews were undertaken by a research assistant not connected with the Emergency Department or local diabetes service (KA), and each interview commenced with an introduction and explanation of confidentiality principles.

Audio-taped interview data were transcribed verbatim into Microsoft Office Word™, deidentified and imported into NVivo^TM^ software before being analysed using Gibbs’s thematic [12] framework. This framework entails transcription and familiarisation, code building, theme development, and data consolidation and interpretation. All comments and responses were treated confidentially. Data were analysed by an experienced qualitative research team member who was independent of the interviews and not aware of the identity of participants (SJ). Findings were discussed amongst all research team members until consensus was reached.

Ethical approval was obtained from the Children’s Health Queensland Hospital and Health Service (HREC/19/QCHC/56600) and University of the Sunshine Coast (A191341) Human Research Ethics Committee. Public Health Act and appropriate site-specific approval were also obtained.

## Results

Interviews were conducted with four patients/representatives (Adults = 3; and Parent of a child = 1) and 18 healthcare professionals from different professions (Physician = 7; Registered Nurse = 6; Pharmacist = 2; Dietitian = 1; and Diabetes Educator = 2) employed across varying healthcare settings, and working at differing levels of expertise and responsibility. Participants had a wide variety of experience working with patients with T1D in DKA, ranging from employment in primary healthcare settings, to diabetes services, Emergency Department resuscitation bays and hospital wards. Interviews lasted a mean 14.6 minutes.

Healthcare professional participants recounted stories of patients with T1D in DKA to whom they had provided healthcare recently. Besides those presenting in DKA immediately prior to a diagnosis of T1D, healthcare workers perceived that patients presenting in DKA were likely to be adolescents or young adults, and that alcohol abuse and homelessness were significant factors.

Describing factors relating to DKA presentations in people with T1D, interventions to support them and for prevention, three themes emerged: access, contributing factors and health system opportunities.

### Theme 1: access

Access and delays in treatment were described as factors affecting people who presented with DKA. Patients’ access to insulin and related administration devices in the community were largely not a concern for patient and healthcare professional participants, partly as a result of Australian Government funding. However, access to ketone testing supplies and continuous glucose monitoring technology was considered by some to pose major barriers to prevention of DKA presentations. This was both in terms of financial cost and, specific to ketone testing supplies, physical access; many reported local pharmacies did not stock test strips.

*“Pretty much most things are subsidised. But when it comes to ketone strips, a pack of ten cost $10. That’s a dollar a strip. I know my brother has T1D and he doesn’t even check his ketones because the strips are quite expensive. It would be nice if they could subsidise them.”* (Patient 2)

The local diabetes education service provided some patients with ketone-testing supplies, although to whom this support was provided was not disclosed. There were, however, limitations on the support that the service were able to offer.

“*Quite often we’ve got a small stock here that we have to hand out to patients just to tide them over to stop them being admitted. So that is a problem …..* .” (Healthcare professional 6)

Access to transport was also perceived as a barrier to prompt DKA presentations. Healthcare professional participants highlighted that many patients were unable to drive, either because they could not afford a vehicle or maintenance costs, or through not having a licence. In explaining why some participants utilise emergency Ambulance services:

“They either don’t have their license, or they cannot afford the gas to come in. “(Healthcare professional 4).

### Theme 2: contributing factors

Patient and healthcare professional participants highlighted factors that they had observed to contribute to, and possibly delay, DKA presentations in these patients. Many of these reasons were arguably preventable. Patients commonly acknowledged, and were regarded as, not always prioritising the management of their T1D and, potentially, subsequent DKA. The purchase and use of insulin did not appear to always take precedence for some patients, possibly influenced by a limited insight into its importance for their health and wellbeing:

“*They’ve got other priorities and insulin isn’t high on it. I don’t think that they understand fully how bad it can be for them if they let their diabetes play up, don’t sort it out properly and don’t keep it under control.*” (Healthcare professional 12)

Patients also had difficulty in planning and organisation for insulin supplies. Occasionally, patients were reported by healthcare professional participants to present to hospital on the day they used their last ampule or pen of insulin, to request another prescription. Further, patients utilising continuous subcutaneous insulin infusion (insulin pump) therapy sometimes didn’t have back-up insulin in case their device malfunctioned. Some patients were thought to be in denial about their medical needs, while others were thought to have made informed choices:

*“A lot of the young people who come in with DKA are perhaps in a bit of denial that they have the severity of T1D.”* (Healthcare professional 13)

Education was deemed by both patients and healthcare participants to be key. However, healthcare professional participants observed that adolescents and young adults diagnosed with T1D as a child appeared to have particularly limited insight. A lack of insight also extended to where parents were separated or where alternate carers were present. Detailing the risks of one parent having limited knowledge around the management of insulin pump therapy, this healthcare professional stated:

*“She* [the patient] *spent the weekend with her dad. She’d been vomiting and was unwell and the dad didn’t know what to do. When the dad was asked how’s she been going, he said that he doesn’t know anything about this. The mother knows how to care for this.”* (Healthcare professional 9)

Other frequently mentioned perceived underlying causes of DKA included social factors, such as alcohol and substance abuse.

### Theme 3: health system opportunities

#### Care provision

Participants highlighted opportunities in the provision of care for patients with T1D around DKA presentations. Hospital-based care for DKA for patients with T1D was, to some extent, according to healthcare professionals, shrouded by mistrust. On the basis of some comments from patients, some diabetes service staff raised concerns around care that patients with T1D receive when presenting in DKA, a potentially life-threatening condition. However, such comments were not always specific to the study hospital.

“*Patients that I have sent over to the Emergency Department to get reviewed have often sat there for a long time before they’ve actually been seen. My patients will report the same type of thing, that families have actually even left before they’ve been seen because they’ve managed to correct it when insulin hasn’t been given when they first presented.*” (Healthcare professional 8)

Further, some patients believed that staff working in Emergency Departments didn’t know a lot about DKA and commented that there was no point in going to hospital because of this. They highlighted a lack of healthcare professional awareness surrounding the acute nature of DKA and down-playing of the patient’s report and experience:

*“I think they need to listen more. Having had it* [T1D] *for twenty-something years it’s like, okay people, I need insulin. So, just that awareness. And then, it took a long time for my insulin infusion to be set up, which obviously led to my sugar levels spiking even more. So, just being aware of what patients are actually saying.”* (Patient 4)

Healthcare professional participants liked the widespread availability of care pathways applicable to patients presenting in DKA, which they had found to be helpful to provide the correct care. With these pathways, Emergency Department staff did not perceive the need for an endocrine specialist to routinely review all patients, and could escalate and de-escalate care as needed. Despite such pathways, concern was highlighted around an expectation of general wards that patients should be completely back to normal before being admitted to the ward: a belief which created a bottleneck in the Emergency Department. Healthcare professional participants working in the Emergency Department expressed concern about the local use of a trial model of triage which didn’t integrate well with the available electronic medical record system. Use of this model had resulted in delays in staff obtaining information that could help determine the presence of DKA, with a subsequently lower triage classification awarded.

Delays in the provision of appropriate and safe care were, however, exacerbated by cultural and language barriers. Two healthcare professional participants reported concern around the translation services available when providing care for patient who did not speak English, instead relying on patients’ accompanying friends and relatives to translate. While phone translations were, depending on the day, considered not too hard to organise, timing translation to be available when needed could be a problem, especially when considering less-common languages. Healthcare professional participants sometimes had to resort to use of Google Translate^TM^.

#### Diabetes educator presence

Concerns were raised by healthcare professionals around the limited reach of current diabetes services, and the impact that this could have on effecting change. For example:

*“Locally, I know that the* [profession] *is on-site at* [the hospital]*, I think it’s two days a week, and they're at* [location] *one day a week and* [location]. *So that one practitioner for the whole of the* [health district] *is spread across three sites in a week. So, my understanding is that it’s quite a limited service.”* (Healthcare professional 14)

Healthcare professional participants highlighted the benefit of having a greater diabetes educator presence in the hospital, as well as in the wider community, providing ongoing education to General Practitioners (GPs) and other primary care staff members; to raise awareness and knowledge of DKA, diabetes in general and clinician management. However, to be successful this would require widespread healthcare professional buy-in:

“*I don’t see any of the other doctors coming down to have education and get upskilled, so there’s a few missing links there I suppose*.” (Healthcare professional 10)

#### Continuity of care and connected care

Participants highlighted opportunities around continuity of care and connected care for patients with T1D, including around DKA presentations. Patient and healthcare professional participants were largely aware of the diabetes support services available within the hospital, with the latter being particularly complimentary of the hospital’s paediatric endocrinology services which provided telephone support outside of regular business hours. However, healthcare participants were largely not aware of external diabetes support services available in the wider community. Where these services were known, some healthcare professional participants, particularly those working in the Emergency Department, were not always sure to whom to refer.

Staff at the diabetes service indicated that they weren’t always aware when someone had presented in DKA:

*“Firstly, we have to know that they’ve actually presented, so that’s probably number one. Quite often we don’t get told that they’re actually - that they’ve actually even presented to Emergency Department and gone home so we don’t even know to follow them up.”* (Healthcare professional 10)

They indicated the benefit of being notified of people who present with DKA to the Emergency Department. They perceived that they may be able to better provide guidance when needed to the treating medical team, and education to the patient about their T1D and DKA management. Concerns were raised, however, by diabetes educators around their ability to consult with patients during a DKA admission in view of staffing and business hours.

The desire for continuity of and connected care amongst participants extended to primary care.

“*I would love to see continuity of care, not fragmented care where the hospital does its bit and says, ‘GP follow-up. This patient’s come here with DKA. We’re discharging them back into your care’*.” (Healthcare professional 3)

GPs were especially viewed as being central to the care coordination of patients with T1D, though concern was raised about their ability to do so due to mechanisms around interprofessional communication, time constraints and available funding. This was compounded by registered patients commonly ‘doctor shopping’, for undisclosed reasons.

*“I just think in general in Australia we haven’t funded GP land well enough to prevent these things coming into Emergency Department. If the GPs were better funded to have more time with each patient and then to refer them onto a nurse and maybe a diabetic educator, then we wouldn’t have these things coming into Emergency Department.”* (Healthcare professional 7)

Both patient and healthcare professional participants did not perceive that patients with T1D were able to access specialised care through a GP, or access care in a timely manner.

To facilitate care continuity and case management between inpatients and outpatients, one healthcare professional suggested weekly or regular meetings with the community-based providers. However, they conceded that this model may not be supported due to budgetary issues whereby acute and community services are funded separately and from different funders.

#### Dedicated telephone support line

The support available to adult patients with T1D after-hours and on non-business days was a concern for most patient and healthcare professional participants.

*“For somebody who’s acutely unwell, if it’s out of our hours then we can’t see them obviously and so they’re going to present to the Emergency Department or they’re going to manage it at home until they’re so acutely unwell that they’re in full blown DKA by the time they get there.”* (Healthcare professional 6)

Concerns were raised by participants about the generic non-specialised information that after-hours support services provided. While detailing the limited attempts to troubleshoot and prevent Emergency Department presentations, this healthcare professional perceived that the only advice generally provided was:

“*If you’re at all concerned please present to your Emergency Department*.” (Healthcare professional 15).

Recognising limitations in the support able to be provided, staff of the adult diabetes service advised patients that during after-hours and non-business days their first contact should be to the Emergency Department if the strategies provided to them were not successful. Detailing a vast attempt to prevent a presentation:

“*Again, I’ve done that. I’ve been on the phone to somebody for the last couple of hours when they’ve gotten to the point where whatever they’re doing is not working and they need some emergency management and I’ve advised that they do present to their local hospital. I’ve even called the hospital for them and spoken to nursing team leader in that department and said, please expect a presentation from this person, this age, this condition and needing support.”* (Healthcare professional 14)

Almost all patient and healthcare professional participants indicated that they believed that a dedicated telephone support line, especially during after-hours and non-business days, should be made available to adults with T1D. This could potentially be shared across more than one provider district, and would prevent many Emergency Department presentations for DKA through the provision of, for example, troubleshooting and sick day management. Highlighting the patient voices:

*“That would be nice, because in the past when we’ve had these issues on weekdays, I’ve been able to call the educators and say, this is what’s happening, do I take her to the hospital or can you suggest anything to do, and they’re able to point me in the right direction and give me advice. That way I can manage it at home rather than coming straight into the hospital. Whereas on weekends and after hours, the support’s not really there.”* (Patient 2)

“I think it would, especially if, say, my parents were able to call them and get advice as to what they might be able to do. There’s certainly an interim time where it could be managed outside the hospital if other people knew how to help me.” (Patient 3)

“I think that would be good, especially in Emergency Departments. That way you do have the access to somebody that actually specialises in that area, because I find a lot just shrug it off because they don’t have the knowledge.” (Patient 4)

Patients and healthcare professionals advocated that this telephone support line should be staffed by diabetes educators, with appropriate authority to provide treatment advice.

## Discussion

Our study has provided insights into patients’ and healthcare professionals’ perceptions of factors affecting presentation of people with T1D to the hospital Emergency Department with DKA, possible interventions that may improve the standard of care and preventative strategies for this patient population. Findings revealed that poor access to ketone testing supplies, continuous glucose monitoring technology and transport were described as system factors in DKA development. Other factors included prioritisation and organisation around the management of T1D, lack of education and social factors, such as alcohol and substance abuse. Opportunities to improve the care available to patients with T1D were detailed, including increased spread of current diabetes services, improvements in healthcare professional DKA knowledge, and patient continuity of care. Almost all participants supported the need for a dedicated telephone support line for adults with T1D, especially after-hours and non-business days.

Socioeconomic factors are widely documented as important determinants of health and wellbeing, in Australia and internationally. Higher financial income, education or occupation levels are associated with higher health, with lower socioeconomic groups at greatest risk of poor health, disability and death [13, 14]. When considering T1D, low socio-economic status, both at and after diagnosis, has been associated with factors such as poor glycaemic control [15], dyslipidaemia [16], hypertension and smoking [17]. Further, low socioeconomic status has been associated with adverse events in children and teenagers utilising insulin pump therapy [18]. It is important to address barriers and facilitate T1D management and timely presentation for any DKA, specifically for people in low socio-economic areas; to continue public health advocacy for investment to address inequities at the level of social determinants.

Restricted access to ketone testing supplies and continuous glucose monitoring technology was described as a factor in DKA development. In Australia, the federal government subsidises the cost of many diabetes-related supplies via the National Diabetes Services Scheme [19]. However, this scheme does not cover ketone-testing strips, though there is some coverage for urine-testing strips. Cover is, however, provided for continuous and flash glucose monitoring technologies, for groups including children and young people with T1D aged under 21 years, and those with T1D over 21 years with concessional status. Considering the financial cost of a DKA presentation [20–22], it would be useful to conduct economic analyses of the costs and gains of subsidised provision of ketone-testing strips, and extension of age-limited concessions for continuous and flash glucose monitoring technologies, especially for families on lower incomes.

Access to transport was also perceived as a barrier to prompt DKA presentations. In Queensland, Australia, emergency Ambulance services are provided free by the State Government [23]. This, and other options for transport to hospital, could be evaluated and compared.

Opportunities to improve the care available to patients with T1D were offered, including details of care provision, employing diabetes educators, models of continuity of care and connected care. Collectively, such findings are not unique to the study hospital. Nurses worldwide have been reported with significant and long-standing knowledge deficits in diabetes care [24]. Knowledge deficits are not unique to T1D, and have also been reported, for example, for chronic obstructive pulmonary disease management [25]. In many locations, improving the care available to patients with T1D would require reconfiguration with appropriate apportionment of resourcing to support care delivery.

Regardless of age, specialist care should be available to people with T1D outside of traditional business hours, for example, for troubleshooting and sick day management and advising on acute care needs to avoid unnecessary use of acute healthcare services [3]. Almost all participants in our study, whether patients or healthcare professionals, believed that a dedicated telephone support line should be made available to adults with T1D, especially outside office hours and business days. Across Australia, the use of telephone support lines is not new, with numerous non-diabetes-specific after-hours telephone health support services available. For example, a Nurse on Call service for general health concerns is available in Victoria [26], and the Rural link service provides after hours’ mental health telephone support for people in rural communities in Western Australia [27]. However, these services are not designed to specifically assist people with T1D, whose queries are usually met with the advice to go to the local Emergency Department. Alternative approaches to ED for 24-h care for people with T1D should be developed and evaluated. After-hours phone support specific to patients with T1D has been provided in sites in Australia; in Western Sydney, for example, it was associated with reduced progression of ketosis to DKA [28]. There is an opportunity to further evaluate such a model, especially in low socio-economic settings, and this should be explored. Such a model may also be beneficial when considering acute complications in other chronic diseases. Further research to determine the benefit of this, together with other opportunities to improve the care available to patients with T1D, is warranted.

Limitations of our study should be acknowledged. Recruitment was by self-selection and as such this may have generated sampling bias; findings may not be representative of all patients and healthcare professionals. Further, healthcare professionals were employed by a single public healthcare provider, and findings reflect their experiences and perceptions at one time point. There was also limited patient data and elucidation of participants’ experience with patients with T1D and DKA; their limited exposure may have influenced their perceptions. Data saturation for patients was not obtained, and as such, conclusions drawn from these interviews should be considered preliminary. Many patients who present and re-present with DKA may perhaps be less likely to participate in a research study, when considering common comorbidities such as mental health [29, 30]. However, strengths derive from varying patients and healthcare disciplines from which data were obtained.

## Conclusions

This research provides important insights into the experiences and perceptions of patients and healthcare professionals with T1D around the topic of DKA. Findings indicate gaps in support for patient self-care to avoid DKA and prevent late presentation of DKA. Service reconfiguration is indicated to support care delivery particularly in relation to avoidance and timely management of this damaging and potentially fatal consequence metabolic disorder. One relatively simple change could address access to ketone testing supplies and continuous glucose monitoring technology via the National Diabetes Services Scheme. With efficacy already demonstrated, another focus should be the formulation and national use of dedicated telephone support lines for adults with T1D, especially outside office hours and business days. Further changes could be developed, tailored to local needs and resource availability. However, until change is made, people with T1D will continue to make both avoidable presentations (incurring unnecessary expense for patients, families, providers and communities) and delayed, acutely unwell, presentations to Emergency Departments.

## Supplementary Information

**Additional file 1.**

## Data Availability

The datasets generated and/or analysed during the current study are not publicly available due to lack of consent and authorisation allowing this. All data generated or analysed during this study are included in this published article [and its supplementary information files].

## Abbreviations

T1D: Type 1 diabetes
DKA: Diabetic ketoacidosis
GP: General practitioner
