# Supplementary material for: Diabetic ketoacidosis presentations in a low socio-economic area: are services suitable?
Source: BMC Health Serv Res. 2021 Jul 10;21:682. doi: 10.1186/s12913-021-06715-7 (PMC8272902; doi:10.1186/s12913-021-06715-7)
Supplement: Supplementary file 1 — Additional file 1. [file 12913_2021_6715_MOESM1_ESM.pdf]

Appendix 1: Semi-structured interview guide

Healthcare consumers

-Can you please tell me about your/your child's recent presentation to the Caboolture Hospital ED for DKA?

-What specialist diabetes services are provided locally for people with T1D?

-Do you attend specialist diabetes healthcare services? Where and why?

-Was difficulty around access to timely acute care healthcare professional support a factor in the presentation?

-Is access to acute healthcare professional support a concern for you?

-Was difficulty around access to insulin a factor in the presentation?

-Is access to insulin a concern for you?

-Are there any other factors that contributed to the presentation?

Are there any possible interventions that you would suggest that may improve the standard of care of people with T1D?

-What would you suggest occur to help prevent the need for a DKA ED presentation specifically?

Healthcare professionals

-Can you please tell me about your professional background?

-Can you please tell me about your experience working with patients with T1D that have presented to the ED in DKA?

-Was difficulty around access to timely acute care healthcare professional support a factor in any presentations?

-What specialist diabetes services are provided locally for people with T1D?

-Is access to acute healthcare professional support a concern for you?

-Was difficulty around access to insulin a factor in any presentations?

-Is access to insulin a concern for you?

-Are there any other factors you perceive may affect the presentation of people with T1D to the Caboolture Hospital ED in DKA?

-Are there any possible interventions that you would suggest that may improve the standard of care of people with T1D?

-What would you suggest occur to help prevent the need for a DKA ED presentation specifically?
